# 

**Published:** 1880-12

**Authors:** 


CONTENTS.

| | Page. | | Page. |
|---|---|---|---|
| Nasal Catarrh | 235 | Can Animals Talk? | 255 |
| Measles and Scarlet Fever | 237 | How a Man Goes to Bed | 256 |
| Diphtheria | 238 | The Pronunciation of "U" | 257 |
| Egyptian Pad | 239 | Asthma Cure | 259 |
| Toothache Drops | 239 | Removal of Stains and Spots | 260 |
| Care of Infants | 240 | Roses | 261 |
| Moles, Warts and Birth-marks | 241 | The William Goat | 261 |
| The Coming Man Physically | 242 | Book Notices | 262 |
| A Remarkable Man | 243 | Perils of Flattery | 264 |
| The Late General Myer—The Signal Service | 244 | Mother and Infant | 268 |
| Poetry | 246, 259 | High Pressure Steam | 271 |
| Life Insurance | 247 | Fever and Ague | 272 |
| Croup | 249 | Diseases of the Spine | 273 |
| Texas | 250 | Colds | 274 |
| Taken Down | 251 | Are Metals Volatile? | 275 |
| Sciatica | 253 | Baking Powders | 276 |
| Goethe and Schiller | 254 | Consumption | 277 |

